# Biomarker Discovery and Redundancy Reduction towards Classification using a Multi-factorial MALDI-TOF MS T2DM Mouse Model Dataset

**DOI:** 10.1186/1471-2105-12-140

**Published:** 2011-05-09

**Authors:** Chris Bauer, Frank Kleinjung, Celia J Smith, Mark W Towers, Ali Tiss, Alexandra Chadt, Tanja Dreja, Dieter Beule, Hadi Al-Hasani, Knut Reinert, Johannes Schuchhardt, Rainer Cramer

**Affiliations:** 1 MicroDiscovery GmbH, Marienburger Str. 1, 10405 Berlin, Germany; 2 Department of Pharmacology, German Institute of Human Nutrition Potsdam-Rehbruecke, Arthur-Scheunert-Allee 114-116, 14558 Nuthetal, Germany; 3 Department Computer Science and Mathematics, Free University of Berlin, Berlin, Germany; 4 Department of Chemistry and The BioCentre, The University of Reading, Whiteknights, Reading, RG6 6AS, UK

## Abstract

**Background:**

Diabetes like many diseases and biological processes is not mono-causal. On the one hand multi-factorial studies with complex experimental design are required for its comprehensive analysis. On the other hand, the data from these studies often include a substantial amount of redundancy such as proteins that are typically represented by a multitude of peptides. Coping simultaneously with both complexities (experimental and technological) makes data analysis a challenge for Bioinformatics.

**Results:**

We present a comprehensive work-flow tailored for analyzing complex data including data from multi-factorial studies. The developed approach aims at revealing effects caused by a distinct combination of experimental factors, in our case genotype and diet. Applying the developed work-flow to the analysis of an established polygenic mouse model for diet-induced type 2 diabetes, we found peptides with significant fold changes exclusively for the combination of a particular strain and diet. Exploitation of redundancy enables the visualization of peptide correlation and provides a natural way of feature selection for classification and prediction. Classification based on the features selected using our approach performs similar to classifications based on more complex feature selection methods.

**Conclusions:**

The combination of ANOVA and redundancy exploitation allows for identification of biomarker candidates in multi-dimensional MALDI-TOF MS profiling studies with complex experimental design. With respect to feature selection our method provides a fast and intuitive alternative to global optimization strategies with comparable performance. The method is implemented in R and the scripts are available by contacting the corresponding author.

## Background

Diabetes mellitus is one of the most common chronic diseases in nearly all countries and subject to intensive biomedical research. The prevalence of diabetes is forcast to increase from 285 million in 2010 to 439 million in 2030 [1]. Diabetes imposes an increasing economic burden on national health care systems world wide as 12% of the health expenditures are anticipated to be spent on diabetes in 2010. The global costs of treatment will raise from 418 billion USD in 2010 to 490 billion in 2030 [2]. The major part of the prevalence is due to obesity related type 2 diabetes (T2DM).

Multiple studies have been performed assessing the diversity of the disease at the transcriptomic level revealing lists of candidate genes and associated pathways [3,4]. At the proteomic level different techniques have been applied including gel-based [5] and mass spectrometry (MS)-based quantitative approaches [6]. However, in most cases the study design is rather simple and restricted to the comparison of healthy versus diseased animal or human samples. No comprehensive proteomics study covering multiple experimental factors and comprising a multitude of samples has been published so far.

In this manuscript we investigate a multifactorial matrix-assisted laser desorption/ionization (MALDI) MS plasma profile data set based on a T2DM mouse model, using NZO (New Zealand Obese) and SJL (Swiss Jim Lambert) mouse strains. The NZO mouse is an established polygenic model for studying obesity-related diabetes as it rapidly develops symptoms of diabetes characterized by early onset obesity, insulin resistance and eventually destruction of insulin-producing pancreatic beta cells [7]. In contrast, the lean SJL mouse strain is resistant to diet-induced obesity and diabetes, presumably due to a mutation in the Tbc1d1 gene that causes elevated lipid use in skeletal muscle [8].

MALDI MS, particularly in combination with time-of-flight (TOF) instruments, is characterized by simplicity, good mass accuracy and high resolution [9] and hence a promising tool in proteomics [10]. It allows for processing a significant number of samples in a short time and therefore enables studies encompassing a multitude of samples [11-13]. MALDI-TOF MS profiling has been used extensively for investigating different types of cancer like breast cancer [14], lung cancer [12,15], ovarian cancer [16] or colon cancer [17,18], to name a few. Biomarker identification and classification are the typical objectives in MALDI profiling studies of disease models. Various different methods have been applied addressing these two objectives. For feature selection commonly used methods comprise the classical t-test or Wilcoxon rank sum test [19] as well as more advanced techniques such as genetic algorithms and swarm based intelligence [20]. With respect to classification Wu et al. [21] published a summary comparing statistical methods for ovarian cancer. In 2006, Zhang et al. [22] compared the performance of R-SVM and SVM-RFE using MALDI MS data sets and more recently, in 2009, Liu et al. [23] compared additional feature selection and classification approaches.

In general, proteomic data has two different types of replications, (1) biological and (2) technical, leading to two different types of errors, and therefore requires proper statistical analysis. The standard approach of handling technical replicates is to calculate a mean value in order to reduce the technical noise. Unfortunately, this can lead to loss of information [24]. A more sophisticated way to handle technical replicates without loss of information are mixed-effects models [25,26]. They incorporate fixed-effects parameters applied to the entire population and random effects applied to particular experimental units or sub-units (e.g. technical replicates). However, for the high number of biological replicates in this study the results for both methods are similar.

**Figure 1:**
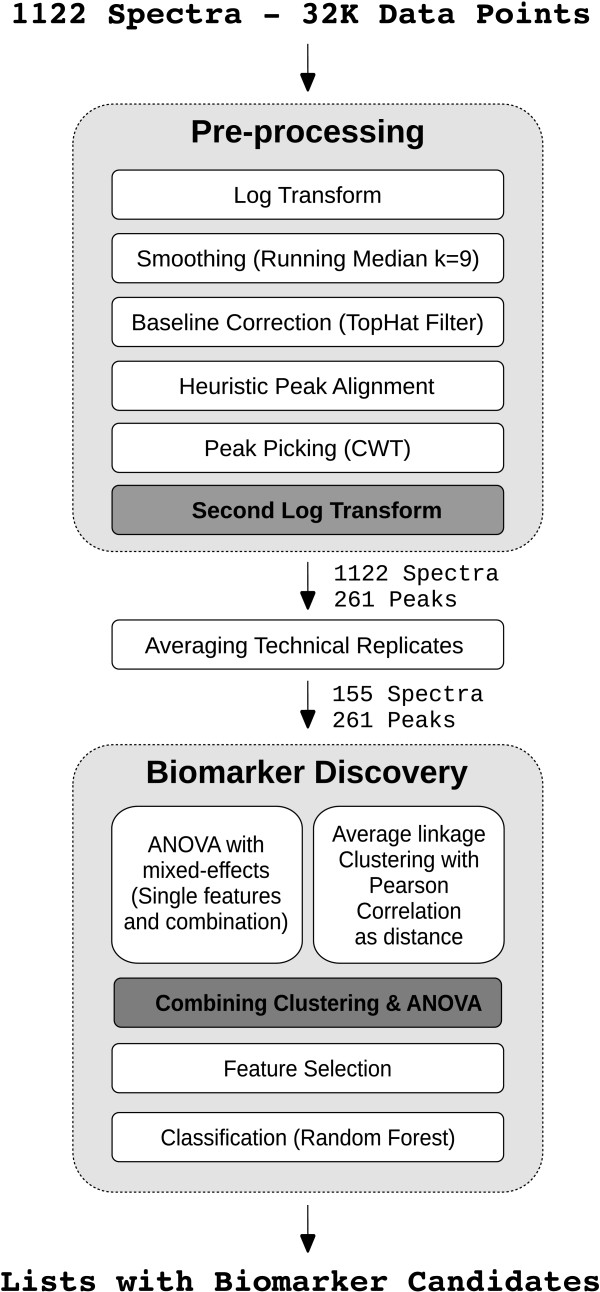
**Work-flow**. Complete work-flow of the cluster-based ANOVA approach with feature selection for multi-factorial MALDI MS profiling data in biomarker discovery.

Although many approaches have been developed for biomarker identification from MALDI MS profile data, only some studies were performed for assessing the influence of correlation in these datasets [27]. As correlation within large MS data sets can confound statistical analyzes, we developed statistical methods that exploit data correlation and integrated these into a comprehensive work-flow designed for the analysis of multi-factorial experimental MALDI-TOF MS data. Merging similarity and significance information our approach allows for the interpretation of complex biological data in an intuitive manner. The soundness of the statistical methods is demonstrated and a special plot for easy visualization and understanding. Furthermore the presented methods provide a natural way of feature selection for classification and prediction. The complete work-flow of the analysis is shown in Figure 1.

## Methods

### Data

The study design involved the experimental factors genotype, diet and time.

#### Genotype

Three different mouse strains were examined: C57BL/6J (B6), NZO (New Zealand Obese) and SJL (Swiss Jim Lambert). The New Zealand Obese mouse strain exhibits polygenic obesity associated with hyperinsulinaemia and hyperglycaemia and presents additional features of a metabolic syndrome, including hypertension, and elevated levels of serum cholesterol and serum triglycerides [28]. NZO mice are highly susceptible to weight gain when fed a high-fat diet, resulting in the development of morbid obesity, with fat depots exceeding 40% of total body weight and the development of type 2 diabetes [29]. In contrast, the Swiss Jim Lambert (SJL) mouse strain is lean and resistant to diet-induced obesity and diabetes [30]. B6 mice represent an intermediary phenotype between NZO and SJL at later age (> week 12) with respect to sensitivity to diet-induced obesity and diabetes. While the genetic and molecular basis for the different diabetes susceptibilities of polygenic mouse strains is largely unknown, we recently identified a naturally occurring loss-of-function mutation in the Tbc1d1 gene in SJL mice that increases lipid oxidation in skeletal muscle and as a result confers leanness and protects from diet-induced obesity and diabetes [8].

#### Diet

After weaning at week 3, male B6, NZO and SJL mice were raised on three different diets, a low fat diet (SD; 8% calories from fat) and two different high fat diets, one containing carbohydrates (HF; 35% calories from fat) the other one a carbohydrate-free (CHF; 72% calories from fat). We have shown previously that HF diet strongly induces insulin resistance and may lead to diabetes, whereas CHF equally induces peripheral insulin resistance but protects from diabetes [7,31]. At week 8, mean body weight of SJL mice was 18.81 g (+/- 1.46 g) on SD, 20.04 g (+/-0.99 g) on HF and 21.24 g (+/- 2.31 g) on CHF. In contrast, mean values for NZO mice were 31.94 g (+/- 1.36 g) on SD, 33.72 g (+/- 4.39 g) on HF and 36.6 g (+/- 4.83 g) on CHF, respectively. Mean values for B6 mice were 20.1 g (+/- 2.56 g) on SD, 20.54 g (+/- 0.78 g) on HF and 22.32 g (+/- 1.38 g) on CHF, respectively.

#### Time

Blood samples were collected at an age of 3, 4, 6 and 8 weeks from the mouse tails.

#### Sample Preparation

Blood samples were obtained by cutting the tip of the mouse tail and collecting the blood from the dorsal and lateral tail veins into a Li-heparin-coated microcuvette. Immediately after blood collection each sample was centrifuged at 4°C for 5 min at 13,000 rpm. The blood plasma was then transferred into 200L-microcentrifuge tubes, shipped on dry ice to the mass spectrometry laboratory and stored at - 80°C prior to further sample preparation and MS analysis.

The amount of plasma obtained at each blood collection varied between 0 and 12 *μl*. Essentially the same procedures were applied as reported previously for the MALDI sample preparation of blood serum samples [16,32], taking into account the partly lower sample volumes available.

Since 5 *μl *were needed for each sample preparation, it was possible to perform up to two sample preparations. In a few cases only one or no sample preparation could be performed. From each sample preparation 4 replicate MALDI MS profile spectra were acquired, resulting in a total of up to 8 technical replicates per sample. The number of samples and spectra for each combination of experimental factors is stated in Table 1.

**Table 1:** MALDI Number of Samples.

		**week 3**				**week 4**				**week 6**				**week 8**		
								
		**SD**	**HF**	**CHF**		**SD**	**HF**	**CHF**		**SD**	**HF**	**CHF**		**SD**	**HF**	**CHF**
								
B6		36/5	31/4	12/2		36/5	40/5	37/5		38/5	38/5	32/5		39/5	34/5	28/5
NZO		35/5	35/5	32/4		40/5	36/5	40/5		37/5	38/5	40/5		28/5	34/5	34/5
SJL		4/1	0/0	16/3		12/2	0/0	40/5		32/4	40/5	32/5		36/5	40/5	40/5

Number of MALDI mass spectra and biological replicates for each factor combination. The first number indicates the number of spectra, the second states the number of biological replicates. In total there are 1122 spectra for 155 different biological samples derived from 31 different mouse individuals.

MALDI MS spectra were obtained using an Ultraflex MALDI-TOF/TOF mass spectrometer (Bruker Daltonics, Bremen, Germany). Spectra were acquired automatically for the m/z range of 700-10,000. MS profile peak identification was achieved similarly to the methods described in reference [33] using a Q-Tof Premier mass spectrometer (Waters, Manchester, UK).

### Pre-Processing

The pre-processing work-flow of the MS data aims at transforming the large number of data points in raw spectral data (typically > 30, 000) into a much smaller, statistically manageable set of peaks. Mass spectrometry data is inherently noisy due to underlying chemical processes (interference from matrix material, sample contamination, degradation) and the physical measurement process [34]. Various algorithms differing in methodology, implementation and performance have been proposed to deal with the noise. Several reviews [35-38] describe and evaluate the pre-processing steps. A widely accepted standard sequence of pre-processing steps is:

1. Log transformation

2. Smoothing

3. Baseline correction

4. Peak alignment

5. Peak picking

A multitude of software packages implementing the complete work-flow is available. Commonly used public domain software tools are R and Bioconductor [39] packages like msProcess or PROcess [40], Matlab packages like LIMPIC [41] or Cromwell [42] and the comprehensive C++ library OpenMS [43,44].

Statistical tests such as ANOVA require intensity data for each feature to be normally distributed and the variance to be independent of the intensity (additive error behavior). We tested different variations of pre-processing methods and finally chose the following procedure leading to stabilized variance: log transformation, smoothing using a median filter (windowSize = 9) and baseline correction with a tophat filter [45] (see Figure 1).

For peak alignment we used a heuristic approach: We began with the identification of 43 reference peaks from the mean spectrum of all 1122 spectra using continuous wavelet transform (CWT) peak picking algorithm [46,47]. Peak picking was performed for each individual spectrum to be aligned. If a peak was found in an environment of 30 index positions around a reference peak we calculated their distance. The distances to reference peaks are constant for a spectrum and thus, the final index shift value for a spectrum is calculated by averaging the corresponding distances (for detailed visualization of index shift values and a pseudo-code notation of the alignment algorithm see Additional file 1: Peak alignment).

Peak picking was done using CWT implemented in Bioconductor [39] package msProcess employing second derivative of a Gaussian function (Mexican Hat Wavelet) as mother function (parameters: *scale.min = 3, length.min = 7, noise.fun = "quantile"*). Although CWT is somewhat complicated and slow, it is very stable against noise due to internal data smoothing and shows good and reliable performance (see Bauer et al. [48] for a detailed evaluation and comparison of different peak picking algorithms). Furthermore, the internal data smoothing of CWT makes the whole pre-processing robust to changes of the smoothing parameters. Using CWT we successfully identified 261 peaks.

**Figure 2:**
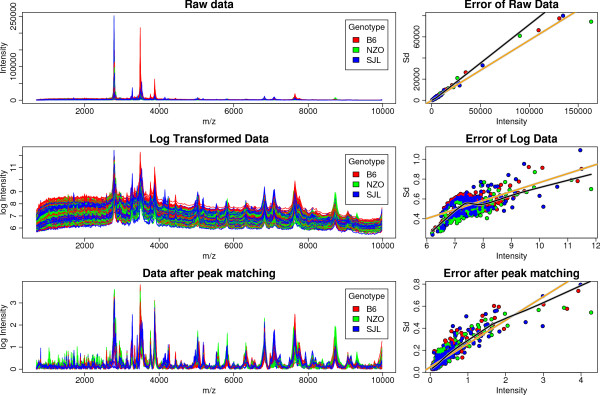
**Preprocessing**. MALDI MD profiling raw data (top), log data (middle) and after baseline correction and peak alignment (buttom). The left column show the effect on the spectra itself while the right column shows the corresponding standard error plots including linear fit (orange line) and lowess fit (black line). The different colors reflect different genotypes (red: B6, green: NZO, blue: SJL).

**Figure 3:**
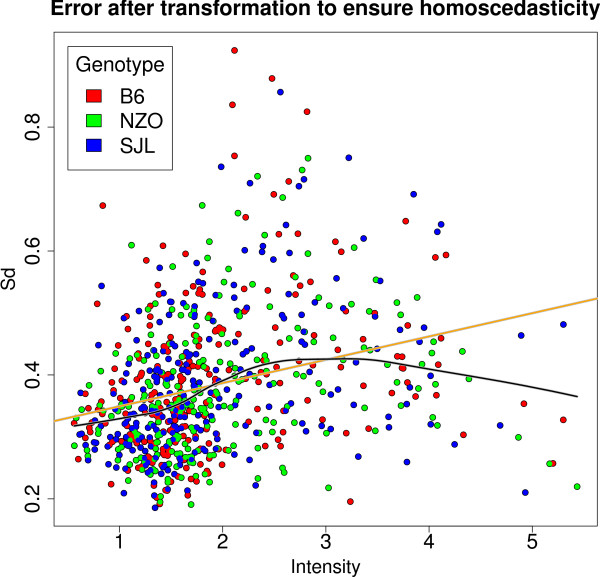
**Error Plot to ensure homoscedasticity**. Error plot after log transformation to ensure homoscedasticity including linear fit (orange line) and lowess fit (black line). The different colors reflect different genotypes (red: B6, green: NZO, blue: SJL).

The effects of log transformation, baseline correction and peak matching are depicted in Figure 2. After applying logarithmic transformation to the spectra the correlation between variance and intensity is still strong. However even the combination of log transformation, baseline correction and peak mapping does not lead to a stabilization of the variance which is necessary for applying our statistical analysis methods. Hence, in order to assure homoscedasticity additional steps were required. Obviously, there is still a linear dependency between variance and intensity indicating a multiplicative error model (see Figure 2). In order to account for this, we applied another log transformation. We added a pseudo-count of 0.1 to avoid the singularity at 0. Finally we added an offset for convenience. After this transformation the data are homoscedastic (see Figure 3).

While the input for the complete pre-processing work-flow consists of 1122 continuous spectra each with 32,000 data points, the output is a list comprising intensities for 261 discrete peak positions for all 1122 spectra (see Figure 1).

Technical replicates are not independent and hence violate an assumption of ANOVA. Because of this, technical replicates were averaged prior to statistical analysis (see Figure 1). By averaging, the 1122 individual spectra were reduced to 155 mean spectra.

### ANOVA

The main idea of ANOVA (ANalysis Of VAriance) [49] is to partition the variance into subcomponents with respect to one or more explanatory variables. The following types can be distinguished: One-way ANOVA, Multi-way ANOVA, and ANOVA with mixed effects model [50].

#### One-way ANOVA

One-way ANOVA is used to test for differences in one variable describing *k *(two or more) independent groups, e.g. multi-stage disease. For *k *= 2 one-way ANOVA is equivalent to the t-test. Let *μ_i _*denote the mean of the *i^th ^*group containing *n_i _*elements then ANOVA tests for the null hypothesis *μ*_1 _= *μ*_2 _= ... = *μ_k_*. If the null hypothesis is rejected than at least two of the means are not equal. The result does not provide any information about how many and which means differ. Performing the corresponding *k *· (*k *- 1)/2 pairwise t-tests would lead to a loss in significance due to the required multiple testing corrections. Using the residuum sum of squares (RSS) of any kind of fitted linear model (describing the variable of interest), ANOVA defines an f-value. Assuming normal distributions within the groups the f-value distribution is now *f *~ *F *(*k *- 1, *N *- *k*) and allows for the definition of a corresponding p-value.

#### Multi-way ANOVA

Multi-way ANOVA analyzes the effects of *d *(two or more) independent variables containing *k_d _*(two or more) independent groups, e.g. analyzing different treatments and various disease states. In contrast to multiple one-way ANOVAs the RSS is calculated from a single model for all variables. Thus, the degrees of freedom and the distribution of the f-values are different which has to be accounted for in the calculation of the corresponding p-values.

#### ANOVA with mixed-effects model

ANOVA with mixed-effects model looks for the effects of several (not necessarily independent) variables and also accounts for the effects coming from combinations of variables, e.g. analyzing the effect of different treatments for various disease states. The underlying model can either relinquish group combinations (model 1 with *p*_1 _parameters) or include group combinations (model 2 with *p*_2 _parameters). If the first model is nested within the second one, the f-value can be calculated as (n = sample size, RSS = Residuum Sum of Squares):(1)

The f value is distributed as *f *~ *F *(*p*_2 _- *p*_1_, *n *- *p*_2_).

### Stratification and Clustering

After pre-processing each peak should represent a peptide or peptide combination (for simplification we focus on the case of one peptide only). The concentration of a peptide varies in the diverse samples (diet-genotype combinations). The list of peptide peak intensities (N = number of samples) will be called intensity profiles within this manuscript.

Due to fragmentation/degradation each protein can split up into multiple peptides and lead to multiple peaks in the mass spectrum. These peaks are not independent and the corresponding intensity profiles are therefore correlated. High correlation between intensity profiles can indicate related peptides as in multimer formations or post translational modifications (PTMs). However, in order to benefit from this kind of correlation or any technical redundancies various methods have been proposed [27]. For this study, we apply hierarchical clustering using average linkage [51] with 1- *ρ *as distance measure, where *ρ *denotes the Pearson-correlation coefficient [52]. Each node in the resulting cluster dendrogram represents several intensity profiles and similar intensity profiles are aggregated in close proximity.

Clustering is a standard tool in data mining but there are only a few studies using clustering in this context (e.g. [53]). A great advantage of our approach is the combination of the similarity information with significance by assigning p-values to the nodes. For the question under consideration the appropriate statistical test like t-test or ANOVA defines a p-value for each leaf. For aggregated nodes based on *n *leafs the p-value is calculated from the mean intensity profile of corresponding peaks. For technical and biological reasons intensity profiles are on different absolute scales. Therefore prior to averaging intensity profiles, they have to be z-transformed [51].

### Classification and Prediction

Proper feature selection is essential for building a classifier that accomplishes good performance without overfitting. One can distinguish three kinds of feature selection methods: filter methods, embedded methods and wrapper methods [20,54]. Filter methods are independent of the classification and do not consider the feature similarity or orthogonality. Embedded methods include the feature selection process in the classification training. Wrapper methods use non-linear global optimization strategies like genetic algorithms or swarm based intelligence approaches. Wrapper methods succeed in optimizing classification results but they also tend to overfitting. Embedded methods require complex algorithm adaptations for most classifiers. Filter methods are straight forward but are often outperformed by the other methods [55].

## Results

### ANOVA with mixed effects

A major goal of this work is the analysis of the mutual influence of diet and genotype on blood proteins within a T2DM study. For the data presented here, a straight forward approach for this analysis was a mixed-effect ANOVA of the form:

This model investigates effects derived from all three single experimental factors as well as the combination of genotype and diet (symbolized by the '*'). Time as a further experimental factor was of minor biological interest during this analysis. The ANOVA analysis was performed as described in the Methods section.

### Average Linkage Clustering

**Figure 4:**
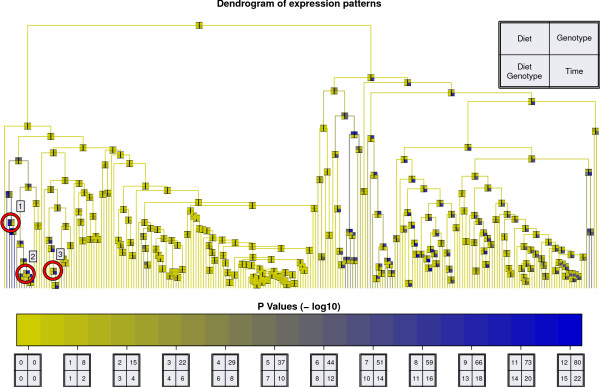
**Cluster Dendrogram**. Cluster dendrogram of all peaks identified in this dataset (see the Methods section for details). Every node is characterized by four ANOVA p-values shown as a color-coded box with four fields: diet (upper left), genotype (upper right), time (lower right) and combination of diet and genotype (lower left). The different -*log*_10 _p-value colorscales for the four factors are shown at the bottom. Three clusters for further discussion (see text) are marked with red circles.

In parallel to ANOVA an average linkage clustering was performed. The cluster dendrogram combining correlated peptides and ANOVA p-values (see Figure 4) was calculated as described in the Methods section. The experimental factors have different impact on the data (see Figure 4). The most significant p-values are obtained for genotype (up to 10 ^-91 ^). The different mouse types can be easily distinguished using the profile data. Diet and the combination of genotype and diet seem to have a much smaller but still substantial effect on the data (p-values of up to 10 ^-14 ^) whereas time has an even greater effect (p-values of up to 10 ^-23 ^). Nearly one third of all peaks - the whole right part of the dendrogram - is associated with the experimental factor time. On this global level the dendrogram allows an intuitive overview of the complete data set as both similarity and significance information are shown in a unified representation.

### Profile Similarity for Hemoglobin

**Figure 5:**
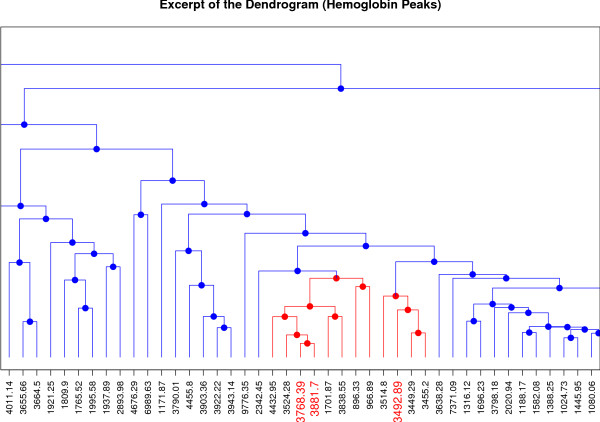
**Dendrogram Hemoglobin**. Excerpt of the dendrogram in Figure 4 showing the three peaks identified as hemoglobin (colored red on the x-axis).

Protein composition of blood is typically dominated by albumin and other highly abundant proteins such as hemoglobin. Albumin and hemoglobin are large proteins represented by a multitude of peptides and thus should be presented by multiple peaks in our dataset. Assuming that many of their peptides are correlated they should be located in close proximity in the dendrogram. MS-based profile peak identification revealed one albumin and three hemoglobin peptides. Mapping the three hemoglobin peptide peaks in the dendrogram shows that they are indeed in close proximity (see Figure 5) verifying our assumption. The peak identified as albumin is located in the big cluster in the central part.

### Identification of biomarker candidates in Multi-Factorial Studies

**Figure 6:**
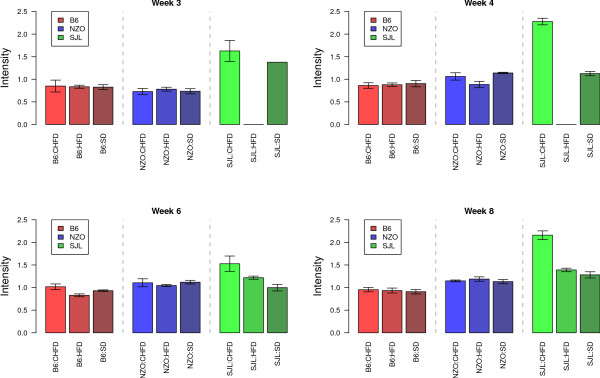
**Peak 4075**. Normalized peak intensities for the peak at m/z 4075 representing cluster 1 of the dendrogram in Figure 4. Peak intensities for all 3 experimental factors are drawn as bar plots with error-of-mean error bars. Genotype and diet are given below the bars for each week. The missing values for the SJL-HFD week 3 and 4 samples are due to the sample collection problems described in the Methods section.

Table 2 provides an overview of the three clusters marked with a red circle in Figure 4 and their corresponding peaks. Each cluster comprises peptide peaks that have been partially analyzed and identified. Cluster 1 comprises three peaks with a mean correlation coefficient of 0.71 and is the most significant result for factor diet (p-value of 10^-10^). Cluster 1 has also the most significant p-value for the combination of diet and genotype (10^-14^) and a p-value of 10^-19 ^for genotype. A detailed illustration of the intensity profile for peak m/z 4075 can be seen in Figure 6. This peak shows high intensities for the combination of SJL-genotype and CHF-diet whereas it is almost constantly low for all other factor combinations. This effect is also visible with lower significance for diet or genotype only. However, only the combination of the two experimental factors assesses the proper biological mutual influence.

**Table 2:** Table for clusters 1-3.

						**p values**										
						
**Cluster**		**mean cor**		**Peak**		**Diet**			**Genotype**			**Week**			**Diet ∗ Genotype**	
												
1		0.71		2262		1.3e-10	0.00015		2e-19	3.8e-11		0.0018	0.97		4.6e-14	1.2e-05
				3618			6.7e-07			2e-08			0.004			8.8e-13
				4075			2e-14			1.5e-29			3e-08			7.9e-14
												
2		0.94		9305		0.96	0.0019		3.3e-31	4.3e-36		2.8e-19	2.4e-17		0.12	0.013
				8720			0.38			1.2e-23			2.8e-16			0.2
				8735			0.57			8.2e-29			5.5e-22			0.21
												
3		0.95		6329		0.0012	0.022		7.5e-75	6.7e-50		0.34	0.24		0.18	0.19
				4237			9.5e-05			1.1e-70			0.61			0.022
				5029			0.00014			1.3e-91			0.82			0.14
				5822			0.0023			5.7e-81			2.3e-07			0.82

Table for clusters 1-3 of Figure 4. For every cluster and peaks aggregated within this cluster, the correlation of the peaks and the ANOVA p-values for the three different experimental factors and the factor combination of Diet and Genotype are given. P-values are given for every peak separately and for the complete cluster.

Cluster 2 consists of three peaks with a mean correlation coefficient of 0.94. The p-values for genotype and week are significant: 10^-31 ^and 10^-19 ^respectively. The intensity is higher in NZO mice and this effect increases during aging while there are only minor differences between the diets.

As already mentioned the genotype has the strongest effect in this dataset. The four peaks of cluster 3 are strongly associated with genotype (p-value of 10^-75^). The mean correlation of the six peaks is 0.95 and they are only present in SLJ genotype mice independent of diet or week. A detailed illustration of the intensity profile for peak m/z 3388 can be seen in Additional file 2: Results for Genotype.

In the middle of the dendrogram (of Figure 4) a big cluster is visible containing 43 peaks of which one has been identified as albumin. All 43 peaks in this cluster have a mean correlation coefficient of 0.84. This cluster is not associated with any of the experimental factors.

All p-values are given without multiple testing correction. Applying rigid Bonferoni multiple testing correction for 261 tests, the p-value threshold of 0.05 changes to 0.05/261 = 0.0002. Hence all p-values discussed above remain significant.

### Classification and Prediction

The methods established in the previous section are also well-suited for obtaining reliable and precise classifications and predictions. This can be demonstrated by the example of diet and the classification performance can be evaluated by cross validation. Thus the task is to predict the diet applied from the data. The other two experimental factors are less suited for purpose of demonstration because genotype classification is rather simple (c.f. Additional file 2: Results for Genotype) and time is sampled from a continuous support and less suited for formulation of a classification task. Using the method described above for feature selection we avoid the shortcoming of typical filter methods as clustering incorporates information about similarity and orthogonality. We found it to be sufficient to use one representative feature from the cluster to achieve classification performance comparable to wrapper methods.

In order to demonstrate the advantages of cluster-based ANOVA we built a classification system with a decision tree based classifier for the experimental factor diet [56]. Since the optimal feature size for classification strongly depends on the classifier and on feature-label distribution [57], we performed classification with different feature set sizes: 3, 5 and 8. The feature selection itself was done three times by selecting top features from:

1. ANOVA analysis without clustering: Selection of peaks with the most significant p-values for experimental factor diet (Peaks m/z: 1883, 3267, 3407, 4075, 4237, 5176, 5536, 8332).

2. Ant colony optimization strategy: Using an ant colony optimization strategy [58,59], we identified a set of features with optimized classification results in a similar way to Ressom et al. [20] with 200 ants and 100 iterations (Peaks m/z: 3267, 3437, 3575, 4041, 4237, 4965, 6569, 7058).

3. ANOVA analysis including clustering: Selection of clusters or peaks with the most significant p-values for the experimental factor diet. For every cluster, the peak with the most significant p-value is selected as representantive for the cluster (Peaks m/z: 1883, 3267, 3407, 3556, 3943, 4075, 5176, 8332).

Confusion matrices for 10-fold cross validation are shown in Table 3 together with a p-value for the classification result calculated by comparing the performance of the selected set of features with the performance of randomly selected sets.

**Table 3:** Confusion Matrices.

				CHF				HF				SD						
												
nFeat		Method		CHF	HF	SD		CHF	HF	SD		CHF	HF	SD		Error		P-Value
3		ANOVA		**33**	14	18		17	**24**	24		16	16	**36**		0.53		0.0028
		ACO		**45**	4	16		10	**33**	22		11	16	**41**		0.4		1e-08
		Cluster ANOVA		**40**	12	13		15	**28**	22		9	16	**43**		0.44		1e-06
												
5		ANOVA		**36**	13	16		18	**22**	25		20	11	**37**		0.52		0.006
		ACO		**48**	3	14		12	**33**	20		10	15	**43**		0.37		2.7e-08
		Cluster ANOVA		**40**	13	12		15	**38**	12		4	24	**40**		0.4		6.7e-07
												
8		ANOVA		**41**	12	12		16	**34**	15		6	22	**40**		0.42		9e-06
		ACO		**45**	5	15		12	**30**	23		4	18	**46**		0.39		5.5e-07
		Cluster ANOVA		**43**	10	12		14	**35**	16		5	19	**44**		0.38		3.3e-07

Confusion matrix for 10-fold cross validation for experimental factor diet using random forest classifier. The feature selection was done by three different methods: ANOVA, ant colony optimization (ACO) and cluster-based ANOVA. The feature selection was performed three times with different number of features: 3, 5 and 8. Numbers in bold print represents true positives.

Using ANOVA without clustering for feature selection leads to a 10-fold cross validation error of 53% for 3 features (p-value: 0.0028), 52% for 5 features (p-value: 0.006) and 42% for 8 features (p-value: 1 · 10^-06^). As expected the ant colony feature selection outperforms the simple filter method with a cross validation error of 40% for 3 features (p-value: 1 · 10^-08^), 37% for 5 features (p-value: 2 · 10^-08^) and 39% for 8 features (p-value: 5 · 10^-07^). However, our improved feature selection technique leads to performances comparable to wrapper method in terms of cross validation errors (44%, 40%, 38% for 3, 5 and 8 features) and p-values(1 · 10^-6^, 7 · 10^-7^, 3 · 10^-7 ^for 3, 5 and 8 features).

## Discussion

The ANOVA model applied analyzes the effects of single experimental factors as well as the combination of diet and genotype. Before applying ANOVA we ensured that all required assumptions are fulfilled (e.g. homoscedasticity and *χ*^2 ^distribution of errors). Hence, ANOVA is the perfect candidate for the statistical analysis and preferable to non-parametric Kruskal-Wallis test since is has greater power.

Peaks identified in the analysis of feature combination provide valuable additional information. For instance, the most significant result found for the combination of genotype and diet was identical with the most significant result for diet (see Table 2). Looking solely at the factor diet we would conclude that peak m/z 4075 is correlated with diabetes-protective CHF diet [7,31]. An analysis of the factor combination, however, shows that this correlation with the CHF diet only stems from the SJL genotype (see Figure 6), which is completely invisible for single factor analysis.

Another interesting outcome of our analysis is the fact that the peak m/z 8735 in cluster two is associated with the growing fat of NZO mice. First it is significantly higher in NZO mice then in the other genotypes that do not develop prominent diet-induced obesity. Secondly it increases with age and thus with body weight of the NZO mice. Therefore the corresponding polypeptide is a candidate biomarker and a potential target towards T2DM disease mechanism. Thus, further in-depth functional analysis of this marker, and its relation to diet-induced obesity and insulin resistance may provide important insights into the pathophysiology of diabetes and its secondary complications.

As seen in the Results section, there is no direct overlap between the top eight features for classification of diet selected with ant colony optimization and the top features selected with ANOVA. However, the best features of ant colony optimization are also characterized by top ranked p-values. Both lists of top 50 features show a rank correlation of 0.5 (Spearman correlation) and hence both lists are not that different. Even the best features have only low discriminative power and as a result there are multiple sets of features leading to similar classification results as seen in Table 3.

In the middle of the cluster dendrogram (Figure 4) there is a cluster having many very good correlated peaks. One possible reason for that is a large common protein being the common source of all those peaks. A perfect candidate for this role would be albumin as it consists of 608 amino acids. This hypothesis is supported by the fact that one of the peaks was indeed identified as belonging to albumin.

Table 4 shows the distinctive properties of our approach compared with other methods. Standard t-test is often the method of choice for statistical testing and the selection of suitable features for classification and prediction. However, standard t-test is not adequate for multi-dimensional datasets since it investigates only one variable with exact two independent groups at the same time. F-test allows for testing multi-dimensional datasets and ANOVA enables to investigate factor combinations. Similarity of features is not considered by any of the statistical tests. Swarm intelligence or genetic algorithms are a different group of algorithms aiming at biomarker candidate identification. Although they are applicable to multi dimensional datasets and take data redundancy into account they often fail in producing deterministic results and p-values. Our work is designed to retain all capabilities of statistical testing while considering feature similarities at the same time. In addition to having similar performances comparable with Swarm intelligence methods, other great advantages of our system are reduced complexity and computational requirements. While feature selection with ant colony optimization took roughly 5 h with both CPUs on a Intel Core2 Duo CPU (2.66 GHz), the cluster based ANOVA took less than 2 seconds.

**Table 4:** Comparison of feature selection methods.

Method	Deterministic	Feature Selection	p-Values	Multi Dimensional	Combinations	Redundancy
t-Test	✓	✓	✓	✕	✕	✕
F-Test	✓	✓	✓	✓	✕	✕
ANOVA	✓	✓	✓	✓	✓	✕
Swarm Intelligence	✕	✓	✕	✓	✓	✓
GA	✕	✓	✕	✓	✓	✓
This Work	✓	✓	✓	✓	✓	✓

Comparison of different methods for biomarker candidate identification and feature selection.

Another advantage of our approach is the possibility to use only one, representative peak from a cluster for further analysis. We have seen that the peaks identified as hemoglobin are located in close proximity in the dendrogram. Hence, we can assume that many of the surrounding peaks are also most likely derived from hemoglobin. Nonetheless, it has to be kept in mind that many peptides originating from the same parent protein will often behave differently. Our approach aims at identifying co-occuring peptides and hence leads to a reasonable reduction of the data. More complex interaction (e.h. high abundance of a protein causes low abundance of another peptide) would require other processing methods, if predominant.

## Conclusion

We have introduced a method that is suitable for identification of biomarker candidates in multi-factorial MALDI-TOF MS profiling studies given an appropriate pre-processing. Applying this method to our data set we were able to identify peaks that are characteristic for the combination of two factors as well as peaks that are significant for single factors. These results are significant even when applying rigid multiple testing corrections. It is shown that ANOVA is an adequate approach for the identification of biologically interesting biomarker candidates from MS profiling data based on multi-dimensional experimental design. Furthermore, classification based on features selected with our approach perform similarly well as those generated with more complex global optimization methods.

## Authors' contributions

CB developed and implemented the described methods and drafted the manuscript. TD, AC and HA were responsible for the generation of the biological samples. AT and CJS acquired the MALDI MS profile data. AT, MWT and RC performed the peptide identification. All authors read and approved the final manuscript.

## Supplementary Material

Additional file 1 **Peak alignment**. Visualization of the results of the peak alignment method. The heuristic algorithm used for peak alignment is presented in pseudo-code. Click here for file

Additional file 2 **Results for Genotype**. Scatter plot of peak intensity values for peaks 3388 and 5029 and peak intensities profile for peak 3388. The peaks are in the list of the most significant results for the experimental factor genotype. Click here for file
