# Supplementary material for: Biomarker Discovery and Redundancy Reduction towards Classification using a Multi-factorial MALDI-TOF MS T2DM Mouse Model Dataset
Source: BMC Bioinformatics. 2011 May 9;12:140. doi: 10.1186/1471-2105-12-140 (PMC3116487; doi:10.1186/1471-2105-12-140)
Supplement: Additional file 1 — Peak alignment. Visualization of the results of the peak alignment method. The heuristic algorithm used for peak alignment is presented in pseudo-code. [file 1471-2105-12-140-S1.PDF]

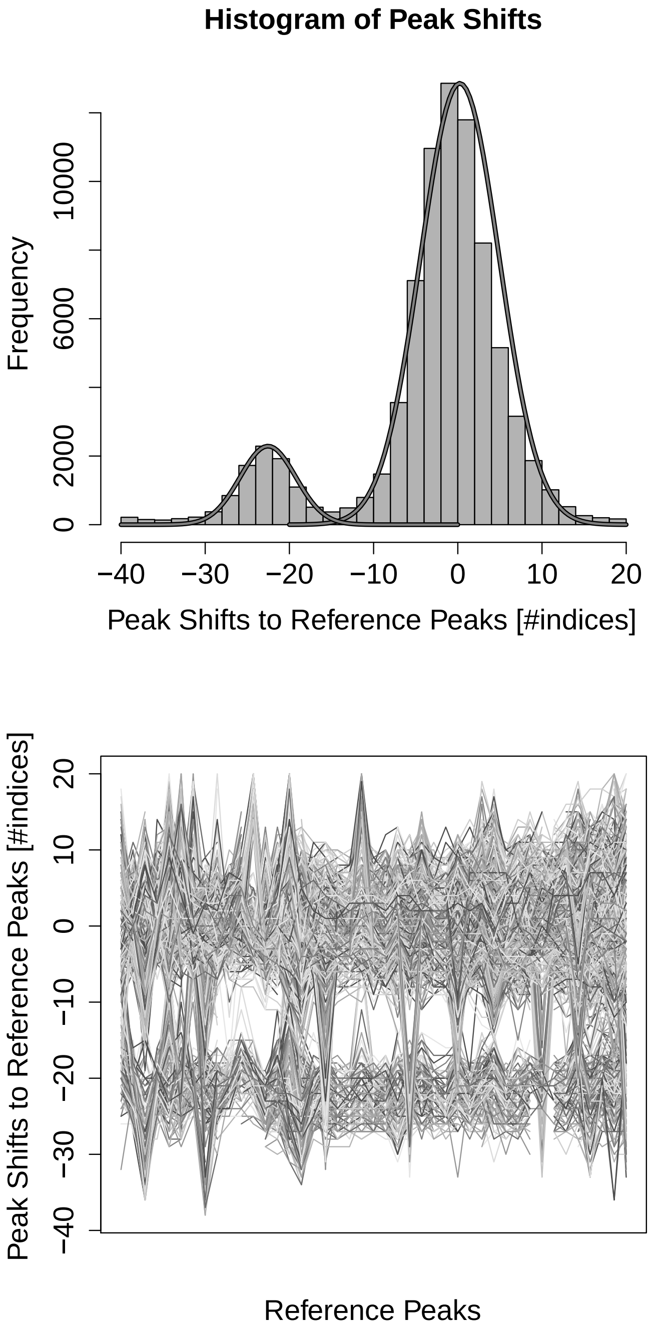

Figure 1: Peak shifts of found peaks and reference peaks calculated during peak alignment process. Left hand side: Histogram of all distances. There are two groups of spectra showing different distribution of peak shifts: one distributes around 0 and one around −22 indices. For both groups a fitted normal distribution is added to the histogram. Right hand side: Vector of peak shifts for the spectra. Again the two groups of spectra are cleary visible. Furthermore for nearly all spectra the shift seem to be constant (on the index scale).

Algorithm 1 Algorithm for peak matching. The peaks are aligned to a reference profile (mean spectrum). The alignment is performed by an index shift.

```
▷ SpectraMat reflects the matrix with spectra (row: sample, col: mass)
function getMeanSpectrum(SpectraMat_{i=1..n,l=1..m})
    RefSpec ← SpectraMat_{1,}
    for i = 2 to n do                                            ▷ every spectrum
        for l = 1 to m do                                        ▷ length of a spectrum
            RefSpec_l ← RefSpec_l + SpectraMat_{i,l}
        end for
    end for
    for l = 1 to m do
        RefSpec_l ← RefSpec_l/m
    end for
    return RefSpec
end function

function getAlignedSpecta(SpectraMat_{i=1..n,l=1..m})
    ▷ calculation reference peaks and distances to reference peaks
    RefSpec ← getMeanSpectrum(SpectraMat)
    ▷ nrp = number of reference peaks (43)
    RefPeakList_{1..nrp} ← PeakPicking(RefSpec)
    Distances_{i=1..n,j=1..nrp} ← NA                             ▷ Store Distances
    for i = 1 to n do                                            ▷ every spectrum
        for j = 1 to nrp do                                      ▷ every reference peak
            peak ← PeakPicking(SpectraMat_{i,j−d..j+d})
            ▷ d reflects a small environment around the peak (d = 40)
            if peak ≠ NA then
                Distances_{i,j} ← peak − RefPeakList_j
            end if
        end for
    end for

    ▷ calculating average of distances for every spectra
    Displacements_{i=1..n} ← 0                   ▷ Displace indices for each spectrum
    maxDis ← 0                                                   ▷ maximal Distance
    for i = 1 to n do                                            ▷ every spectrum
        for j = 1 to nrp do                                      ▷ every reference peak
            Displacements_i ← Displacements_i + Distances_{i,j}
        end for
    end for
    for i = 1 to n do                                            ▷ every spectrum
        Displacements_i ← Displacements_i/npr
        if Displacements_i > maxDis then
            maxDis ← Displacements_i
        end if
    end for

    ▷ index shift according to displacements
    AlignedMat_{i=1..n,l=1..(m+2·maxDis)} ← NA
    for i = 1 to n do                                            ▷ every spectrum
        for l = 1 to m do                                        ▷ length of a spectrum
            AlignedMat_{i,l+maxDis+Displacements_i} ← SpectraMat_{i,l}
        end for
    end for
    return AlignedMat
end function
```
