# Supplementary material for: Biomarker Discovery and Redundancy Reduction towards Classification using a Multi-factorial MALDI-TOF MS T2DM Mouse Model Dataset
Source: BMC Bioinformatics. 2011 May 9;12:140. doi: 10.1186/1471-2105-12-140 (PMC3116487; doi:10.1186/1471-2105-12-140)
Supplement: Additional file 2 — Results for Genotype. Scatter plot of peak intensity values for peaks 3388 and 5029 and peak intensities profile for peak 3388. The peaks are in the list of the most significant results for the experimental factor genotype. [file 1471-2105-12-140-S2.PDF]

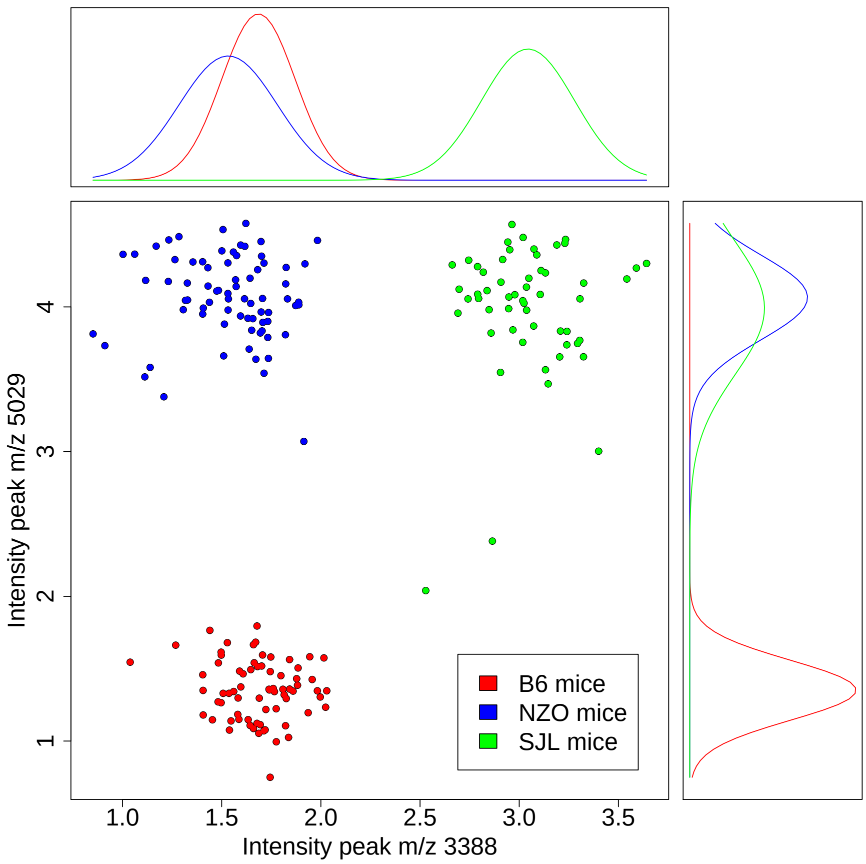

Figure 1: Scatterplot of peaks intensity values for peaks 3388 and 5029. On top and the right hand side are fitted normal distributions for every genotype. Peak 3388 separates SJL genotype from the other two and peak 5029 distinguishes B6 from SJL and NZO. The combination of both peaks allows for a perfect separation of all three genotypes.

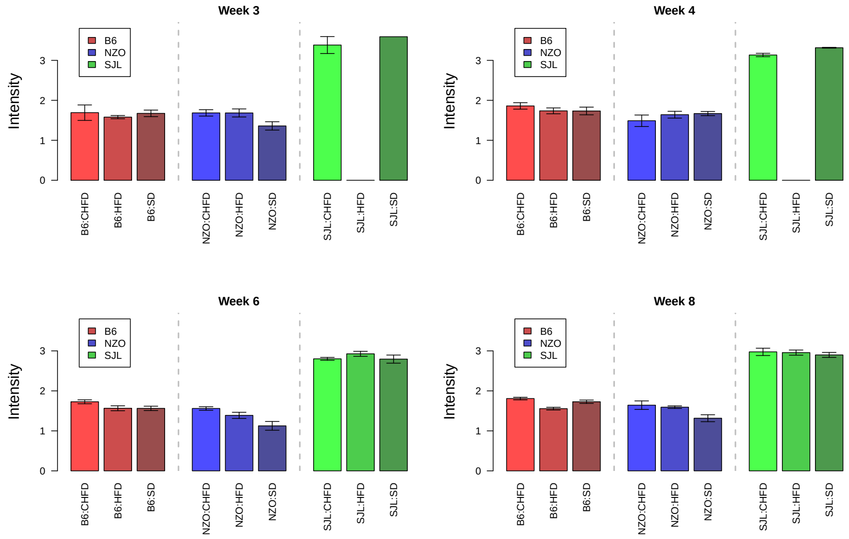

Figure 2: Normalized peak intensities for peak 3388. Peak intensities for all 3 experimental factors are drawn as bar plots with error of mean error bars. Genotype and diet are given below the bars for each week. The ANOVA method is not sensitively affected by the missing values due to sample harvesting problems occuring in SJL-HF in week 3 and 4.
